# Orchiectomy or androgen receptor blockade attenuates baroreflex-mediated bradycardia in conscious rats

**DOI:** 10.1186/1471-2210-6-2

**Published:** 2006-01-23

**Authors:** Gregg R Ward, Abdel A Abdel-Rahman

**Affiliations:** 1 Department of Pharmacology, The Brody School of Medicine at East Carolina University, Greenville, NC, 27858, USA

## Abstract

**Background:**

Previous studies have shown that testosterone enhances baroreflex bradycardia. Therefore, conscious unrestrained rats were used to investigate the role of the androgen receptor in the testosterone-mediated modulation of baroreflex bradycardia. Androgen depletion (3 weeks), and androgen receptor blockade (20–24 h), were implemented to test the hypothesis that testosterone influences baroreflex bradycardia via its activity at the androgen receptor in male rats. Phenylephrine (1–16 μg kg^-1^) was used to assess baroreflex bradycardia.

**Results:**

Androgen depletion attenuated baroreflex bradycardia (P < 0.01). The antiandrogen flutamide (5, 15, or 30 mg kg^-1^, s.c.) caused dose-related attenuation of baroreflex bradycardia in spite of a significant (P < 0.05) increase in serum testosterone. The latter did not lead to increased serum 17β-estradiol level.

**Conclusion:**

The data suggest: 1) Androgen depletion or adequate androgen receptor blockade attenuates baroreflex bradycardia. 2) The reflex increase in serum testosterone may counterbalance the action of the lower doses (5 or 15 mg kg^-1^) of flutamide. 3) The absence of a change in serum 17β-estradiol rules out its contribution to flutamide action on baroreflex bradycardia.

## Background

Most of the research concerning the effects of gonadal hormones on the baroreflex bradycardia has focused on 17β-estradiol. However, our preliminary findings [1], confirmed later by El-Mas et al. [2], have provided evidence that androgens (including testosterone) play an important role in the mediation of baroreflex bradycardia. Reported findings including ours [2-4] have shown that testosterone depletion attenuates baroreflex-mediated bradycardia and that a restoration of baroreflex-mediated bradycardia occurs subsequent to testosterone replacement.

Although the effect of androgens on baroreflex bradycardia has been previously reported, no studies have investigated the possible involvement of the androgen receptor in mediating the effect of testosterone on baroreflex bradycardia. Therefore, flutamide, a relatively lipophilic nonsteroidal competitive central and peripheral androgen receptor blocker devoid of mixed agonist/antagonist effects [5-8] was used in the present study. Notably, there are two actions that must be considered when flutamide is administered. First, it leads to significant elevation in serum testosterone due to its ability to increase plasma levels of lutenizing hormone (LH) via preventing the activation of the testosterone-mediated negative feedback influence on the hypothalamic-pituitary axis [6,9]. Second, it is possible that the increase in serum testosterone will result in an increase in 17β-estradiol, due to aromatization of testosterone to 17β-estradiol via the enzyme aromatase [10]. Like testosterone, 17β-estradiol enhances baroreflex bradycardia in male rats [11]. Flutamide causes an increase in plasma dihydrotestosterone (DHT) in male Sprague-Dawley (SD) rats [12]. The increase in serum testosterone compromises flutamide clinical efficacy [6,9]. Nonetheless, the ability of flutamide to penetrate the blood brain barrier [8], its relatively pure antiandrogenic activity [5], and its similar affinities for the androgen receptor in various androgen-sensitive tissues [14], make this drug a useful agent in investigating the role of the central and peripheral androgen receptor in androgen-mediated responses. In addition, previous research has shown that a gender difference exists in the baroreflex-mediated bradycardia in young rats [15]. Male rats exhibited a greater vagal outflow to the heart in response to baroreceptor activation versus age-matched female rats [15]. Testosterone's contribution to the vagal outflow and not to the sympathetic outflow to the heart of male rats was confirmed by El-Mas et al. [2].

Hence, the main objective of this study was to test the hypothesis that androgens act via the androgen receptor to enhance the baroreflex sensitivity (baroreflex bradycardia) in male rats. The hypothesis was tested by determining whether a) androgen depletion causes an attenuation of baroreflex bradycardia, b) androgen receptor blockade by the competitive antiandrogen, flutamide (5, 15, or 30 mg kg^-1^, s.c.), produces a dose-dependent inhibition of baroreflex bradycardia, and c) the reflex increase in testosterone, which follows androgen receptor blockade [6,9] leads to an increase in serum 17β-estradiol. The studies were undertaken in conscious instrumented rats to avoid the confounding effects of anesthesia [16].

## Results

### Effect of androgen depletion on baroreceptor reflex control of heart rate

**Figure 1:**
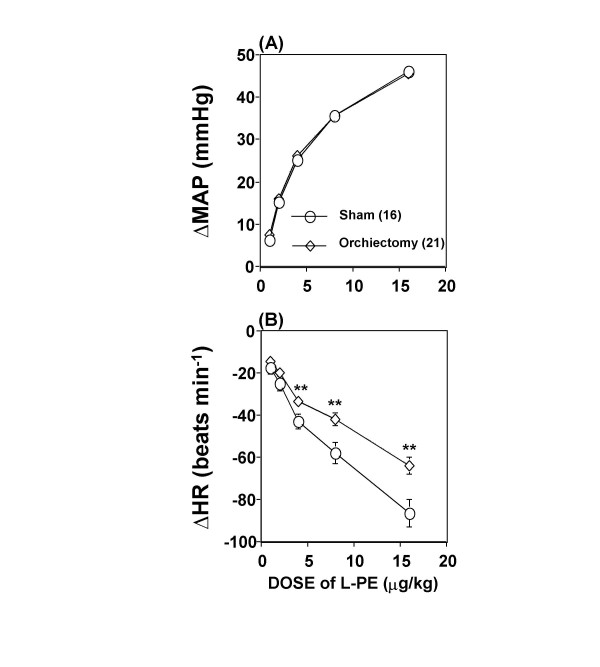
**Dose-Response curves subsequent to androgen depletion**. Dose-Response curves relating increments in mean arterial pressure (MAP) evoked by phenylephrine (L-PE) **(A) **to decreases in heart rate (HR) **(B) **in conscious unrestrained orchiectomized and sham-operated (Sham) rats. Data are means ± SEM. Numbers in parentheses are number of observations. Student's t-test, ** P < 0.01 vs. sham-operated.

**Figure 2:**
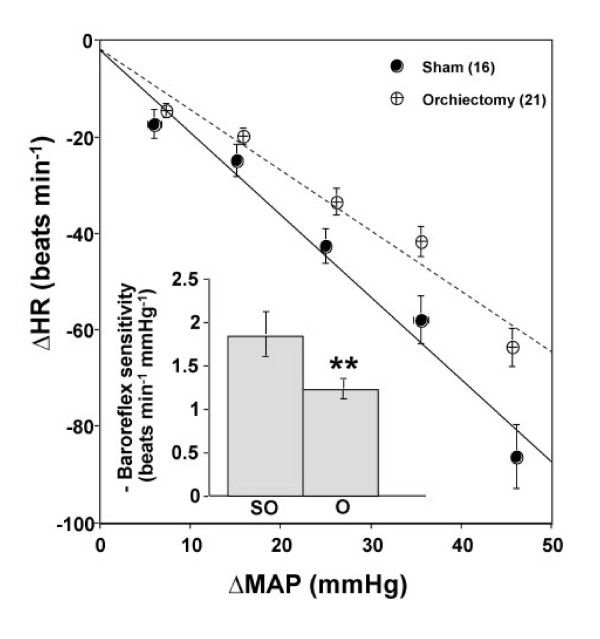
**Effect of androgen depletion on baroreflex sensitivity**. Baroreflex curves relating decreases in heart rate (HR) to increments in mean arterial pressure (MAP) evoked by phenylephrine and baroreflex sensitivity; beats min^-1 ^mmHg^-1 ^in conscious unrestrained orchiectomized (O) and sham-operated (SO) rats. Data are means ± SEM. Numbers in parentheses are number of observations. Student's t-test, ** P < 0.01 vs. sham-operated.

Baseline mean arterial pressure and heart rate values were similar between the sham-operated and orchiectomized rats (mean arterial pressure – sham: 113 ± 1.6 vs. orchiectomized: 109 ± 2.1 mmHg; heart rate – sham: 420 ± 10 vs. orchiectomized: 434 ± 7 beats min^-1^; P > 0.05). All baseline values were obtained on the day of the experiment. Phenylephrine elicited similar rises in mean arterial pressure in all groups (Figure 1A). However, at any given rise in blood pressure, the reflex bradycardic response was significantly attenuated in castrated rats (P < 0.01, Figures 1B and 2). Therefore, the baroreflex bradycardia was significantly reduced compared with the sham-operated rats (-1.23 ± 0.11 vs. -1.84 ± 0.24 beats min^-1 ^mmHg^-1^; Figure 2). This represented an approximately 30% reduction in baroreflex bradycardia. Serum testosterone levels declined subsequent to orchiectomy (9.0 ± 0.4 vs. 487 ± 94.1 ng/dl, P < 0.0001).

### Effect of androgen receptor blockade on baroreceptor reflex control of heart rate

**Figure 3:**
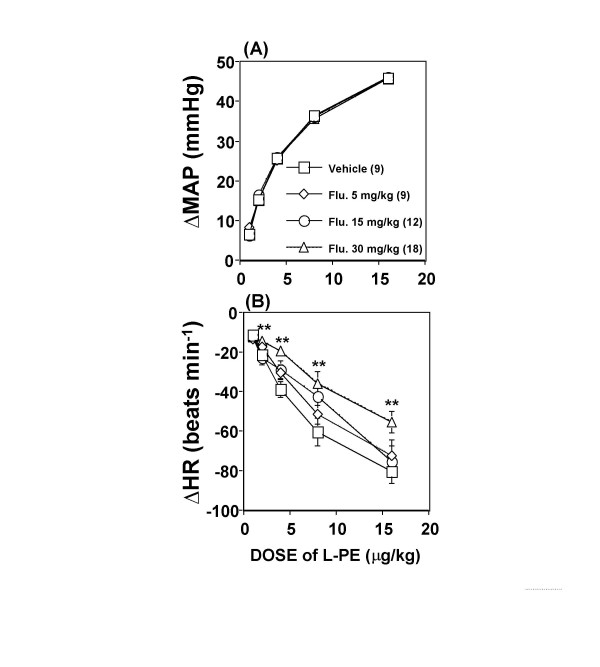
**Dose-Response curves subsequent to androgen receptor blockade**. Dose-Response curves relating increments in mean arterial pressure (MAP) evoked by phenylephrine (L-PE) **(A) **to decreases in heart rate (HR) **(B) **in conscious unrestrained male rats treated with flutamide (Flu.: 5, 15, or 30 mg/kg) or vehicle 20–24 h earlier. Data are means ± SEM. Numbers in parentheses are number of observations. One-Way Analysis of Variance (ANOVA) followed by Fisher's Least Significant Difference post hoc analysis, ** P < 0.01 vs. vehicle.

**Figure 4:**
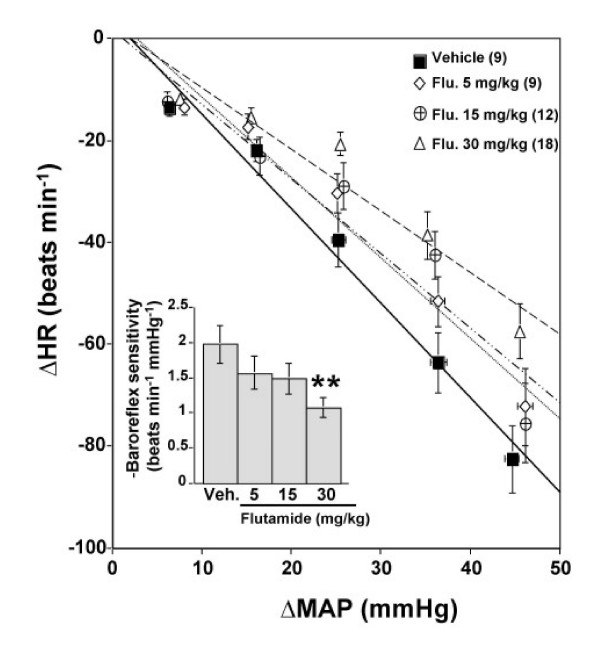
**Effect of androgen receptor blockade on baroreflex sensitivity**. Baroreflex curves relating decreases in heart rate (HR) to increments in mean arterial pressure (MAP) evoked by phenylephrine and baroreflex sensitivity (beats min^-1 ^mmHg^-1^) in conscious unrestrained male rats treated with flutamide (Flu.: 5, 15, or 30 mg/kg) or vehicle (Veh.) 20–24 h earlier. Data are means ± SEM. Numbers in parentheses are number of observations. One-Way Analysis of Variance (ANOVA) followed by Fisher's Least Significant Difference post hoc analysis, ** P < 0.01 vs. vehicle.

**Figure 5:**
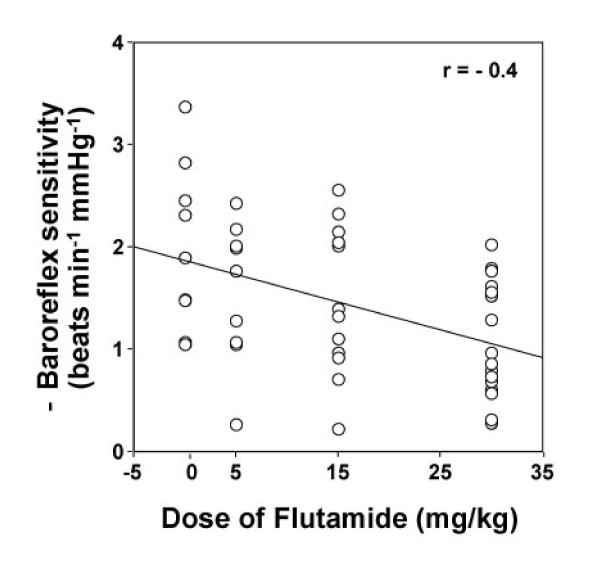
**Inverse relationship between dose of flutamide and baroreflex sensitivity**. Relationship between flutamide dose (5, 15, or 30 mg/kg, administered 20–24 h earlier) and baroreflex sensitivity (beats min^-1 ^mmHg^-1^) in conscious unrestrained male rats.

**Figure 6:**
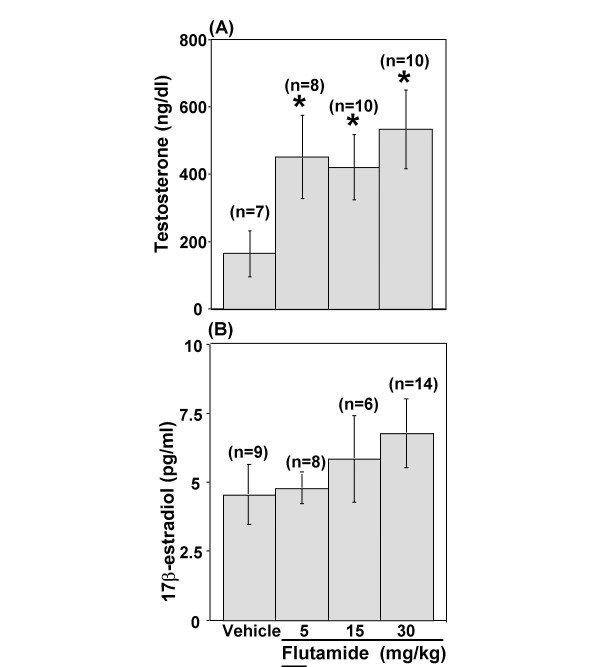
**Effect of androgen receptor blockade on testosterone and 17β-estradiol level**. Testosterone levels **(A) **and 17β-Estradiol levels **(B) **subsequent to flutamide (5, 15, or 30 mg/kg) treatment in conscious unrestrained male rats. Data are means ± SEM. Numbers in parentheses are number of observations. One-Way Analysis of Variance (ANOVA) followed by Fisher's Least Significant Difference post hoc analysis, * P < 0.05 vs. vehicle.

Baseline values of mean arterial pressure and heart rate obtained on the day of the experiment were similar for the treatment and control groups (Table 1). The effect of flutamide (5, 15, or 30 mg kg^-1^, s.c.) on baroreflex bradycardia was assessed 20–24 h after drug administration. Phenylephrine elicited similar rises in mean arterial pressure in all groups (Figure 3A). However, only the highest dose of flutamide produced a significant (P < 0.01, Figures 3B and 4) attenuation of the baroreflex-mediated bradycardia (-1.07 ± 0.14 vs. -1.97 ± 0.27 beats min^-1 ^mmHg^-1^, P < 0.01, Figure 4); a reduction of approximately 45%. Although the reduction elicited by the 2 lower doses of flutamide did not reach statistical significance, (5 mg kg^-1^: -1.57 ± 0.23, and 15 mg kg^-1^: -1.48 ± 0.21 vs. vehicle: -1.97 ± 0.27 beats min^-1 ^mmHg^-1^, P > 0.05; Figure 4), a significant inverse correlation (r = -0.4, P < 0.05, Figure 5) was observed between baroreflex bradycardia and all doses of flutamide. In all treatment groups, flutamide elicited significant (P < 0.05) rises in serum testosterone, which were not dose-related (Figure 6A). Treatment with flutamide did not change serum 17β-estradiol (P > 0.05; Figure 6B).

**Table 1:** Mean arterial pressure (MAP) and heart rate (HR) values obtained in conscious rats 20–24 h following flutamide (5, 15 or 30 mg/kg) or equal volume of vehicle. Data presented as means ± SEM.

**Group**	**n**	**MAP (mmHg)**	**HR (beats min^-1^)**
**Vehicle**	9	105 ± 4	459 ± 19
**Flutamide (5 mg/kg)**	9	111 ± 4	456 ± 14
**Flutamide (15 mg/kg)**	12	105 ± 2.6	465 ± 12
**Flutamide (30 mg/kg)**	18	104 ± 2.7	460 ± 8

## Discussion

The current study presents three new findings. First, androgen receptor blockade by the competitive antiandrogen flutamide attenuates baroreflex sensitivity (baroreflex bradycardia), which suggests the involvement of the androgen receptor in the androgen-mediated enhancement of baroreflex bradycardia in sexually mature male rats. Second, the associated reflex increase in serum testosterone caused by flutamide, which competitively inhibits flutamide activity at the androgen receptor [6,9], may have counteracted the effect of the lower doses of flutamide on baroreflex bradycardia. Third, serum 17β-estradiol level did not change in spite of the significant elevation in serum testosterone in flutamide-treated rats, which rules out the involvement of 17β-estradiol in counteracting flutamide's influence on baroreflex bradycardic responses.

We report here, for the first time, that flutamide attenuates baroreflex bradycardia, which suggests the involvement of the androgen receptor in the androgen-mediated facilitation of baroreflex bradycardia. In a preliminary study [1] later confirmed by El-Mas et al. [2], we determined that androgen depletion produced a 30% reduction in baroreflex bradycardia, which suggests that androgens are necessary for the maintenance of baroreflex bradycardia in sexually mature young male rats. The consequence of androgen receptor blockade on baroreflex bradycardia was investigated to elucidate a mechanism for the previously determined androgen-mediated facilitation of baroreflex bradycardia in male rats [2]. Remmers et al. [21] showed that androgen receptor blockade can be achieved in rats 20–24 h after flutamide administration. Therefore, a similar treatment regimen was adopted in addition to using 3 doses of flutamide (5, 15, or 30 mg kg^-1^, s.c.) to determine a possible dose-dependent effect via the androgen receptor on baroreflex bradycardia. A significant inverse correlation was observed between baroreflex bradycardia and the doses of flutamide, suggesting that the higher the dose of flutamide, the greater the reduction in baroreflex-mediated bradycardia. The substantial reductions in baroreflex bradycardia observed following androgen receptor blockade and androgen depletion were not significantly different. It is notable, however, that the findings of the present study pertain to androgen modulation of the bradycardic component of the cardiac baroreflex response. Whether androgen receptor blockade affects similarly or differently the tachycardic response (tested by nitroprusside) remains to be investigated.

Some evidence, although controversial, suggest that androgens may confer some cardiovascular protection [22-26]. Consistent with this view are the present findings that androgens enhance baroreflex bradycardia via the androgen receptor. The flutamide-induced attenuation of baroreflex bradycardia, if continued on a chronic basis, may lead to hypertension, because in some models of hypertension, an attenuated baroreflex sensitivity plays a contributory role [27]. In effect, some reports indicate that chronic flutamide treatment can produce cardiovascular problems [22], including hypertension [24] in some prostate cancer patients. By contrast, Ganten et al. [28] and Reckelhoff et al. [29] demonstrated an antihypertensive effect for flutamide in young male spontaneously hypertensive rats. In these reported studies, the changes in blood pressure resulted from chronic flutamide treatment, while in the present study a single dose of flutamide was administered to normotensive rats.

The lower doses of flutamide failed to inhibit baroreflex bradycardia, which may be attributed to an associated reflex increase in serum testosterone and 17β-estradiol. The flutamide-induced increase in serum testosterone [6,9], as well as the higher affinity of testosterone for the androgen receptor [14], enable testosterone to compete with flutamide at the androgen receptor. Therefore, the reflex increase and the higher affinity of serum testosterone may have counteracted the effect of the lower doses of flutamide on baroreflex bradycardia. Contrary to our findings, Wichmann et al. [30] reported no increase in serum testosterone subsequent to flutamide treatment although they used similar doses (10 or 25 mg kg^-1^, s.c.) and a similar dosing regimen (every 24 h for 3 days). The discrepancy between both studies may be attributed to their use of male mice and/or their collection of blood samples after euthanasia [30].

Serum 17β-estradiol derived from testosterone [10] enhances baroreflex bradycardia in male rats [11], and may have counteracted the inhibitory action of flutamide on baroreflex bradycardia. However, the absence of an increase in 17β-estradiol after a single dose flutamide argues against a role for this hormone in the present findings. Notably, 17β-estradiol elevation occurred following chronic (≥15 days) administration of flutamide [12,31,32].

The effect of serum dihydrotestosterone (DHT) (derived from the conversion of testosterone by the enzyme 5α-reductase) on baroreflex bradycardia has not been investigated in rats thus far. Serum DHT has a higher affinity for the androgen receptor than testosterone [13]. In addition, an increase in serum DHT has been demonstrated (like serum testosterone) subsequent to flutamide treatment in male rats [12], which may also explain the absence of an effect of the lower doses of flutamide on baroreflex bradycardia. Previous studies [2,3] have demonstrated that the attenuation of baroreflex bradycardia subsequent to testosterone depletion is reversed by testosterone replacement. In addition, we confirmed this finding [4]. This provides strong evidence in favour of testosterone-mediated enhancement of baroreflex bradycardia. Nonetheless, the effect of serum DHT on baroreflex bradycardia needs to be investigated in future studies.

The site of testosterone action on baroreflex bradycardia remains unknown. However, the ability of flutamide to penetrate the blood brain barrier [8], the presence of androgen receptor mRNA in the brainstem regions which control the baroreceptor reflex in male rats [33] and the presence of androgen receptor protein in similar regions in male rats [34], suggest that testosterone's effects may be centrally mediated.

## Conclusion

The present findings suggest that the attenuation of baroreflex bradycardia by castration or androgen receptor blockade involves the interaction between androgens (including testosterone) and the androgen receptor. Androgens may enhance baroreflex bradycardia via the androgen receptor at the level of the baroafferents, the central nervous system or the heart. However, this remains to be determined. Flutamide-induced inhibition of baroreflex bradycardia if continued on a chronic basis, may explain hypertension observed in flutamide-treated patients [24]. A significant reflex increase in serum testosterone, but not 17β-estradiol, seems to limit the ability of the lower doses of flutamide to produce significant attenuation of baroreflex bradycardia. These findings highlight a major role for the androgen receptor in the mediation of the baroreceptor reflex heart rate response.

## Methods

### Preparation of the rats

Male Sprague-Dawley rats (Harlan Farms, Indianapolis, IN) were used in this study. Arterial blood pressure was measured according to the method used in our previous studies [17]. Briefly, the rats were anesthetized with methohexital sodium (50 mg kg^-1^, i.p.). Catheters (polyethylene 10 connected to polyethylene 50), filled with heparinized saline (100 U ml^-1^), were placed in the abdominal aorta and vena cava via the femoral artery and vein for measurement of blood pressure and i.v. administration of drugs, respectively. The catheters were inserted about 5 cm into the femoral vessels and secured in place with sutures. Finally, the catheters were tunnelled s.c., exteriorized at the back of the neck between the scapulae, and plugged by stainless steel pins. Incisions were closed by surgical staples and swabbed with povidone-iodine solution. Each rat received a s.c. injection of buprenorphine hydrochloride (Buprenex; 0.3 μg rat^-1^) to control pain and an i.p. injection of 50,000 U kg^-1 ^of penicillin G benzathine and penicillin G procaine in an aqueous suspension (Durapen) and was housed in a separate cage. The experiment was started 48 h later, which involved the connection of the arterial catheter to a Gould-Statham pressure transducer (Oxnard, CA). The blood pressure was displayed on a Grass polygraph (model 7D, Grass Instruments Co., Quincy, MA). Heart rate was computed from blood pressure wave-forms by a Grass tachograph and was displayed on another channel of the polygraph. Blood samples were collected prior to each experiment for the analysis of serum testosterone and 17β-estradiol levels.

Experiments were performed in strict accordance with institutional animal care and use guidelines, and in accordance with the principles and guidelines of the National Institutes of Health Guide for the Care and Use of Laboratory Animals.

### Orchiectomy

Bilateral orchiectomy was performed as described in previous studies [18] and according to the protocol of the Department of Comparative Medicine, East Carolina University. Under methohexital sodium (50 mg kg^-1^, i.p.) anesthesia, a small surgical incision was made in the center of the scrotum. Each testicle was exposed through the surgical orifice. The ductus deferens and main arteries and veins were isolated, ligated, and severed allowing the testicle and epididymis to be removed. The incision was then closed, sutured and swabbed with povidone-iodine solution. The sham operation involved the exposure of the testes without isolation. The post-operative procedure was implemented as previously described. Finally, the rats were housed in separate cages and allowed free access to food and water.

### Radioimmunoassays

The commercially available radioimmunoassay "Coat-A-Count Total Testosterone" and "Double Antibody Estradiol" kits were used for the analysis of serum testosterone and 17β-estradiol, respectively, and were purchased from Diagnostic Products Corporation (Los Angeles, CA).

### Protocols and experimental groups

#### Effect of androgen depletion on baroreceptor reflex control of heart rate

Two groups of rats (orchiectomized 3 weeks earlier at 250–275 g; orchiectomized: n = 21 and sham: n = 14) weighing 325–350 g at the time of the experiment, were used to investigate whether androgen depletion attenuates the baroreflex heart rate response in conscious unrestrained rats. The rats were allowed to acclimatize to laboratory conditions for at least 2 h prior to experimentation. After connecting the rat to the pressure transducer, blood samples (0.6 ml) were collected, from the femoral artery, for measurements of serum testosterone level. A period of 30 min was then allowed for further stabilization of blood pressure and heart rate. Baroreflex curves were constructed in all rats by the i.v. bolus injection of randomized doses of phenylephrine (1–16 μg kg^-1^) (a pressor agent) at 5-min intervals. Phenylephrine was dissolved in saline and administered in varying volumes of a stock concentration (36 μg ml^-1^) of phenylephrine to achieve the desired doses. Each experiment lasted approximately 1 h. The peak changes in mean arterial pressure and heart rate, obtained following phenylephrine injections, were used for the construction of the baroreflex curves.

#### Effect of androgen receptor blockade on baroreceptor reflex control of heart rate

The rats in each of 3 groups (5 mg kg^-1^: n = 9, 15 mg kg^-1^: n = 12, and 30 mg kg^-1^: n = 18, Table 1) weighing 325–350 g, received one of 3 doses of the competitive antiandrogen flutamide (5, 15, or 30 mg kg^-1^, s.c.), 20–24 h prior to the experiment. The control group (n = 9, Table 1) received an equal volume of the vehicle (sesame oil). Baroreflex bradycardia was assessed as previously described.

### Drugs

Phenylephrine hydrochloride (Sigma Chemical Co., St. Louis, MO), flutamide (Sigma Chemical Co., St. Louis, MO), sesame oil (Sigma Chemical Co., St. Louis, MO), methohexital sodium (Eli Lilly and Company, Indianapolis, IN), buprenex (buprenorphine hydrochloride; Rickitt & Colman, Richmond, VA), povidone-iodine solution (Norton Co., Rockford, IL), and Durapen (penicillin G benzathine and penicillin G procaine; Vedco, Overland Park, KS) were purchased from commercial vendors.

### Statistical analysis

Values are expressed as means ± SEM. The relationship between increases in mean arterial pressure (**Mean Arterial Pressure = diastolic pressure + one-third {systolic – diastolic pressures}**) and associated decreases in heart rate was assessed by regression analysis for individual animals as described in our previous studies [17]. The regression coefficient (slope of the regression line) expressed as beats min^-1 ^mmHg^-1 ^was taken as an index of baroreflex bradycardia [17]. Analysis of variance (ANOVA) followed by Fisher's Least Significant Difference post hoc analysis was used for multiple comparisons. The Student's t-test was used in the analysis of unpaired data, with the level of significance set at P < 0.05. In accordance with reported criteria, control rats possessing (i) baroreflex bradycardia values with significant (P < 0.05) correlation coefficients greater than or equal to 0.8 [19], and (ii) serum testosterone levels greater than or equal to 60 ng/dl [20] were included in the data analysis.

## Authors' contributions

ARA conceived of the study and, along with GRW, participated in the design and coordination of the study as well as the drafting of the manuscript. GRW carried out all of the experiments and the statistical analyses. All authors read and approved the final manuscript.
